# Personality and Behavioral Inhibition/Activation Systems in Behavioral Addiction: Analysis of Binge-Watching

**DOI:** 10.3390/ijerph20021622

**Published:** 2023-01-16

**Authors:** Giuseppe Forte, Francesca Favieri, Maria Casagrande, Renata Tambelli

**Affiliations:** 1 Department of Dynamic, Clinical Psychology and Health, Sapienza University of Rome, 00185 Rome, Italy; 2 Body and Action Laboratory, IRCCS Santa Lucia Foundation, 00179 Rome, Italy; 3 Department of Psychology, Sapienza University of Rome, 00185 Rome, Italy

**Keywords:** personality, binge watching, behavioral inhibition system, behavioral activation system

## Abstract

Binge-watching (BW) refers to a pattern of watching TV series characterized by the consecutive viewing of three or more episodes in one sitting. Although there is some evidence about its effects on mental health, little is known about predictive variables which may affect negative occurrences of BW, such as problematic and addictive behavior. This study aimed to assess the unique contribution of personality traits (i.e., neuroticism, extraversion, agreeableness, openness, and conscientiousness) to binge-watching, while also considering the role of two motivational systems: the behavioral inhibition system and the behavioral activation system. Cross-sectional data from 790 respondents were collected using standardized questionnaires evaluating: BW, personality traits, and the behavioral inhibition/activation system. The possible predictive roles of these variables were tested via hierarchical linear regression models. Our results underline a predictive high-risk role of neuroticism and the behavioral inhibition system and a protective role of conscientiousness in the continuum of BW from a leisure activity to a problematic one.

## 1. Introduction

The development, improvement, and adoption of on-demand streaming platforms services (e.g., Netflix, Infinity, or Amazon Prime) continue to increase yearly. This increase allows unlimited access to a wide array of content and a new pattern of TV series consumption. This phenomenon is known as binge-watching (i.e., watching multiple TV series episodes in one session) [1,2,3].

Concurrently with this expansion, a growing body of research has explored the phenomenology and correlates (e.g., personality characteristics, psychological conditions, and both positive and negative outcomes) of binge-watching, generating an emerging area of scientific inquiry and promoting debates regarding how to define, assess and conceptualize this new media-related behavior [1,2,3].

Some researchers have conceptualized excessive binge-watching as a potentially addictive behavior (e.g., [4,5]) due to similar features of the substance use disorders in both symptomatology (e.g., high tolerance; [4]) and outcomes (e.g., alteration of sleep patterns, impaired behavioral response; [6,7,8]). However, this conceptualization may generate the risk of overpathologizing leisure activities such as the viewing of TV series [3,9]. There is often a tendency to overlook the distinction between intensive—but healthy—involvement in rewarding activities and intensive and problematic involvement [10]. Accordingly, to avoid this mistake, and in line with our previous evidence, we consider the BW pattern in a continuum from a pleasurable to compulsive activity.

Binge-watching shows a dual nature in terms of its associated outcomes [1,11]. Its face of leisure activity is associated with positive mental outcomes, characterized by low stress and anxiety levels and generally high quality of life (Favieri et al., 2022; Forte et al., 2022). Previous evidence also supported the presence of positive outcomes regarding social experience [12,13], enjoyment [7,14], and engagement in positive relationships (e.g., the intensity of prosocial relationships is positively associated with the levels of empathy with fictional characters and narrative transportation; [15]). The negative face of BW, characterized by a problematic pattern with features similar to other addictions, is associated with symptoms of depression and anxiety (e.g., [3,16], unpleasant emotional states, maladaptive coping or emotion regulation strategies (e.g., [1,11,17,18]. Additionally, social withdrawal typically characterizes severe forms of binge-watching. Given this debate, it is essential to enhance our understanding of the transition from binge-watching as a passion or a positive non-interfering engagement to an excessive and uncontrolled behavior with addictive characteristics.

Besides the technological advances facilitating the viewing of TV content (Internet connections, streaming services, etc.) and cultural trends [19], some personological aspects could predict the development of compulsive behavioral patterns associated with modern tools. The development of problematic behavior is considered a consequence of interactions between neurobiological and psychological predisposing variables (e.g., stress management, personality traits, psychopathologies such as depression) and moderating/mediating variables such as affective aspects (e.g., craving, motivation to experience pleasure or to reduce negative mood), cognitive aspects (e.g., reward expectancies, coping style, implicit cognitions), executive functions, and decision making. Accordingly, a wide variety of personological characteristics might make individuals sensitive to binge-watching [3,20].

A recent study by Starosta et al. [16] indicated that specific personality traits such as low conscientiousness, low emotional stability, and low agreeableness are strongly associated with symptoms of BW as addiction. Moreover, the authors speculated on the involvement of the motivational components, suggesting the role of the behavioral driving system in the BW pattern. However, despite the interesting insight, it would be interesting to understand the role of the motivational system in activating and inhibiting behavior in the BW phenomenon in the attempt to solve the question: “What characteristics cause a person to engage in problematic BW behaviors?”. Moreover, little is known about the differences in personological patterns between problematic and non-problematic binge watchers, which is useful for understanding the keys of the continuum. In this sense, personality profiles and motivational behavioral responses can help to outline the risk factors with equal exposure, as highlighted in other addictions or maladaptive behaviors. For these reasons, in a structured effort to progress in a non-pathologized direction, the present article aims at providing personological characteristics and inhibition/activation motivational systems associated with both positive and problematic binge-watching.

## 2. Materials and Methods

### 2.1. Participants

Seven hundred eighty-eight young adults participated voluntarily in the study (mean age = 24.60; SD = 4.64; 64.34% females) by completing an online survey.

For a descriptive purpose, in accordance with the cut-off of the Binge-Watching Addiction Questionnaire (Forte et al., 2021), the respondents were classified into three groups: (1) non-viewers of TV series (answer to the preliminary question “Do you watch TV series?” = NO and score on the BWAQ = 0; N = 24; 3.04 percent of the sample); (2) users of BW as a leisure activity (score on the BWAQ ≤ 50; N = 748; 94.8 percent of the sample); (3) moderately and problematic binge-watchers (score on the BWAQ ≥ 51; N = 16; 2.03 percent of the sample).

### 2.2. Questionnaire

Binge-Watching Behavior: The Binge-Watching Addiction Questionnaire (BWAQ; [3]) was adopted to assess BW behavior. The BWAQ is composed of 24 items on a 5-point Likert scale (i.e., from 0 = never to 4 = always). Some examples of BWAQ items are as follows: Item 2, Item 9, Item 14 and Item 17.

It measures different components of the addictive nature of BW, such as cravings, dependency, anticipation, and avoidance. Moreover, it provides a global score. To define moderate or problematic BW behavior, the BWAQ reports different cut-offs of ≥51 for moderate and ≥69 for problematic BW. The BWAQ reported good reliability in the Italian population (Cronbach’s α = 0.94; [3])

Big five personality: The Big Five Inventory (BFI; [21]; Italian Version: [22]) was adopted to measure personality traits. It is a 44-item inventory that measures an individual on the big five factors (dimensions) of personality [23] on a 5-point Likert scale (1 = disagree a lot; 5 = agree a lot); an example of its items are as follows: Each of the factors is then further divided into different personality facets: extroversion vs. introversion (extroversion), agreeableness vs. antagonism (agreeableness), conscientiousness vs. lack of direction (conscientiousness), neuroticism vs. emotional stability (neuroticism), openness vs. closedness to experience (openness to experience). The Italian validation of the BFI reported good internal reliability of the instrument, with a Cronbach’s α ranging from 0.77 to 0.81 in the different samples included [22].

Behavior Motivation System: The BIS/BAS Scale ([24]; Italian validation: [25]) is a 24-item self-report questionnaire designed to measure two motivational systems on a 4-point Likert scale (1 = very true for me; 4 = very false for me; an example of its items are as follows: 1. the behavioral inhibition system (BIS), which corresponds to motivation to avoid aversive outcomes; 2. the behavioral activation system (BAS), which corresponds to motivation to approach goal-oriented outcomes. Four subscales were included in the instrument, including one subscale to assess BIS and three subscales assessing the three components of BAS: drive (the motivation to follow one’s goals), reward responsiveness (the sensitivity to pleasant reinforcers in the environment), and fun-seeking (the motivation to find novel rewards spontaneously). The Italian validation [25] reported good reliability of the instrument (Cronbach’s α for the BIS and BAS, respectively, of 0.72 and 0.74) with similar results to the original validation [24])

### 2.3. Procedure

A cross-sectional online survey, including questionnaires to assess binge-watching behavior (BWAQ), motivation (BIS/BAS), and personality traits (BFI), was adopted to collect data from the general Italian population, with a dissemination plan to target young adults as a convenience sample. The survey was online from April 2022 to June 2022. Before filling out the survey, participants were informed about the general aim of the study, and they had to fill in an informed consent. No personal information, which could allow the identification of participants, was collected to guarantee anonymity. It was requested that participants allot about 20 min to complete the survey. The overall procedure was approved by the ethical committee of the Department of Dynamic and Clinical Psychology (“Sapienza” University of Rome; protocol number: 0000801) and conformed to the Helsinki Declaration.

### 2.4. Statistical Analysis

Means and standard deviations of continuous variables and frequency and percentage of categorical variables were included in a descriptive session of the results to show the main characteristics of the sample.

Hierarchical linear regression analyses were conducted considering two predictive models (model 1: behavioral inhibition/activation system assessed by BIS/BAS; model 2: behavioral inhibition/activation system assessed by BIS/BAS and personality traits (assessed by BFI) on the global score of BW behavior and on the subscales of the BWAQ assessing the characteristics of the BW pattern (craving, dependency, anticipation, and avoidance). Significance was set at *p* < 0.05.

## 3. Results

The results of the sample classified according to BWAQ scores are reported in Table 1.

### Hierarchical Linear Regressions

Different hierarchical regressions were adopted on a global score of the BWAQ and its subscales, considering as model 1 the two motivational systems assessed by the dimensions of the BIS/BAS questionnaire, and with model 2 also including the personality traits assessed by the BFI.

The results regarding the global score of the BWAQ indicated that both model 1 (R^2^ = 0.01; F4,783 = 3.77; *p* = 0.005) and model 2 (R^2^ = 0.08; F5,778 = 7.47; *p* = 0.001) were significant. Table 2 reported the specific coefficients of the models.

Considering the BWAQ subscales, both the models (craving = model 1: R^2^ = 0.02, F4,783 = 3.52, *p* = 0.007, and model 2: R2 = 0.08, F5,778 = 7.42, *p* = 0.001; dependency = model 1: R2 = 0.02, F4,783 = 3.99, *p* = 0.003, and model 2: R2 = 0.08, F5,778 = 7.03, *p* = 0.001) for craving and dependency. Conversely, model 1 was not significant (anticipation: R2 = 0.01, F4,783 = 2.11, *p* = 0.07; avoidance: R2 = 0.01, F4,783 = 1.96, *p* = 0.09) for anticipation and avoidance, while model 2 was significant for both the subscales (avoidance: R2 = 0.05, F5,778 = 4.30, *p* = 0.001; R2 = 0.04, F5,778 = 3.64, *p* = 0.001). As reported in Table 3, each personality characteristic assessed by BFI had a different role in the features of the BW pattern.

## 4. Discussion

Against the background of the study on BW, the main aim of the present work was to enhance our understanding of the psychological and personality processes underlying binge-watching. By assessing whether motivation and personality traits are associated with the expression of this novel behavioral pattern, we further investigated the characteristics of the continuum of BW from leisure to problematic behavior [3]. This study provided the involvement of the motivational system, inits component of activation and control, along with the unique contribution of personality traits (i.e., neuroticism, extraversion, agreeableness, openness, and conscientiousness) to binge-watching.

Within this framework, our results furnished interesting insight, suggesting a predictive high-risk role of alteration in the motivational inhibition system, confirming the involvement of high levels of neuroticism. Moreover, the protective role of high conscientiousness was highlighted.

The behavioral inhibition system (BIS) is a neuropsychological system that predicts sensitivity to punishment. High levels of BIS activation imply a proneness to loss avoidance and a tendency to display a blunted response to reward [26]. The BIS inhibits behavior that might lead to negative outcomes [27] and presumably explains whether individuals approach potentially rewarding targets or inhibit or withdraw their behavior because of the associated risks [28]. Accordingly, it appears to be directly associated with negative and/or pathological behaviors, such as addiction [29,30,31,32,33,34,35,36]. Moreover, in terms of behavioral addiction, a growing body of literature reported that a high BIS is associated with internet and smartphone addictions [37,38].

Our results suggest a predictive role of the BIS, and not of the BAS, in binge-watching. The BIS independently influences potentially addictive TV series watching, confirming previous findings on other BAs [39,40,41]. This result could be explained by hypothesizing that a highly activated BIS could cause the inhibition of ongoing behavior and subsequent reward-seeking behaviors to avoid aversive cues. In this sense, TV series watchers with a high BIS may use TV series to avoid actively negative moods or daily problems; this pattern could lead to addictive watching behavior.

Regarding personality traits, our results underline the inverse role of neuroticism (as a risk factor) and conscientiousness (as a protective factor). It is well known that personality traits have a relevant role in different addictions, as reported largely from the literature on drugs and substance abuse [42] and recently confirmed in studies on behavioral addictions [40]. For example, it was reported that narcissism is a higher risk factor for social network addiction [43], and extroversion may be a potential risk for both social network and shopping addictions [44,45], while introversion is a possible risk for food addiction [46]. Moreover, previous studies reported that conscientiousness and agreeableness were negatively related to smartphone and internet addictions [47], suggesting that a balance in personality traits would encourage the positive expression of behaviors, while the maladaptive expression of personality traits is associated with problematic and addictive behaviors.

Different personality profiles predict diverse expressions of behavioral addictions. Accordingly, highlighting that personality traits also influence BW behavior in its association with motivational dimensions may help to understand the facets of this phenomenon better, as suggested by the preliminary study by Starosta and Izydorczyk [2].

Starosta and Izydorczyk [2] showed that motivation to deal with loneliness and entertainment motivation associated with conscientiousness, intellect, and agreeableness are the strongest factors related to BW. Similar results were suggested by Tóth-Király, Bőthe, Tóth-Fáber, Hága, and Orosz [5]. Our results confirm and extend these findings. The motivation to cope with loneliness and escape, obtained through TV series watching, may assimilate to the behavioral pattern highlighted by the BIS, delineated by a compromised inhibitory system that enacts dysfunctional behaviors when coping with stressful events, both internal and external. The enactment of these behaviors, involving and affecting both mental health and physiological activation [3], again delineates the close interconnection between mind and body [48].

This aspect could be particularly relevant to preventing a possible behavioral addiction in BW. In fact, some behaviors may be enacted either to escape from a source of stress and out of the inability to control and balance at the homeostatic level. However, some maladaptive behaviors can also be due to the failure to understand one’s own bodily responses, such as tiredness, sleepiness, etc., because the viewer is refuging in another virtual body represented by his/her favorite character of the TV series. Although suggestive, this aspect represents an inference that should be further investigated. This hypothesis should be compared to other possible explanations related primarily to managing stressful events via easily accessible behaviors nowadays, such as access to streaming platforms. If other problematic behaviors emerged in the past as a response to the alteration of the motivation system and expression of personality traits, today, the spread and availability of TV series content may furnish new behavioral patterns. Moreover, the target population of young adults is most likely to benefit from these services.

An interesting point of view of this empirical work is the partial and suboptimal understanding of what happens when behavior that was once healthy and positive becomes problematic. TV series viewing is always motivated by cultural, social, and personological characteristics. However, in some cases, the motivation comes as a consequence of a suboptimal environment, which could be a major risk factor for the development of addiction.

Summarizing the results of this work and comparing the present findings with previous evidence on the continuum of mental health and BW patterns [3], our results confirm the predictive role of motivational and personality factors in adopting BW behavior to different degrees. Moreover, it should be considered that this evidence also emerged in the distribution of BWAQ scores, which presents a lower percentage of problematic BW behavior (2 percent of the sample) than in the general population [3]. This would suggest that the increase of alteration in BW features (i.e., craving, dependency, anticipation, and avoidance) would be predicted by motivational and personality traits in an at-risk behavioral frame, starting from the first and leisure stage of the activity.

However, despite the novel insight, some limits should be highlighted. First, the predictive suggestion should be further supported by the analysis of the longitudinal trend in BW and its expression as both a leisure and problematic BW activity, as well as with the aim to discriminate and furnish an “at-risk personality” across the lifespan. The cross-sectional nature of this work limits the inference on causative roles, as well as if it involves relatively stable individual characteristics.

Another limit is the self-reported nature of the survey, which may also generate response bias that is hard to control for web recruitment. Although recent empirical research largely adopted online surveys, the results should be interpreted carefully. Third, the respondents resulted in an unequal gender and age distribution, with a higher proportion of females and young participants, and an unbalanced group size, especially considering problematic binge-watchers and no-TV series watchers. Although this distribution is in line with previous studies, the results could be less representative of the general population, and further studies should investigate gender and age differences. This aspect represents a relevant limit of the study, which affects the low effect size reported by the regression model (as indicated by the R^2^ index), which may suggest the involvement of other variables in the BWAQ scores, which further studies should consider. Finally, for the exploratory nature of the study, a few exclusion criteria were fixed, which might have affected our findings. Future research on the relationship between problematic binge-watching and mental health and its potential mechanisms is requested to overcome these limitations for understanding this subject better. Moreover, it would be interesting to check other possible behaviors that may be promoted or influenced by similar motivational and personality characteristics, aiming to clarify the expression of behavioral addiction further.

## 5. Conclusions

This work provides another step toward understanding addictive behavior, particularly involving TV series watching. Our society has changed, and the way of watching TV series has also changed; for this reason, observing a new behavior with an old lens does not make sense. We need to consider the new behavioral addictions (i.e., BW), avoiding their pathologization and arriving at the feeling of something already seen for each new behavior that can be, in some way, associated with compulsivity. On the one hand, this study provides data regarding this specific behavioral pattern. On the other hand, it also outlines some characteristics, in this case, negative, which may be associated with multiple expressions of problematic and addictive behaviors often associated with a number of psychophysical consequences and, for this reason, worthy of attention and caution.

Finally, the evidence of this study should be considered in the current frame of the pandemic experience, which reported an increase in a sedentary life, social media use, and TV content viewing. Considering that the COVID-19 pandemic was also related to a significant rise in mental breakdowns [49], it would be interesting to further analyze how personality traits and the executive system affected the behavioral outcomes as a reaction to a negative experience, generating an increased risk of BW addiction.

## Figures and Tables

**Table 1:** Descriptive data of the sample.

	Non-Viewers of TV Series	BW as Leisure Activity	Problematic BW
N (F/M/other)	24 (16/8)	748 (477/261/10)	16 (14/2/0)
Age	24.21 (4.78)	24.63 (4.66)	23.87 (3.24)
BIS/BAS			
BAS_Drive	12.08 (2.78)	11.63 (2.37)	11.68 (2.41)
BAS_FanSeeking	11.75 (2.52)	11.61 (2.26)	11.19 (1.90)
BAS_Reward	17.58 (2.75)	16.76 (2.98)	16.56 (3.81)
BIS	21.62 (4.34)	21.46 (4.32)	22.00 (3.84)
BIG-5			
Conscientiousness	32.25 (8.12)	32.02 (6.25)	28.25 (6.27)
Openness	35.95 (8.50)	36.59 (7.42)	35.81 (7.73)
Extroversion	25.37 (6.93)	25.14 (6.73)	25.75 (5.45)
Neuroticism	23.37 (7.19)	25.81 (5.97)	27.87 (5.40)
Agreeableness	32.54 (6.62)	33.14 (4.68)	32.31 (5.94)

BAS_Drive: motivation to follow one’s goals; BAS_Fun seeking: motivation to find novel rewards spontaneously; BAS_Reward Responsiveness: sensitivity to pleasant reinforcers in the environment. BIS: behavioral inhibition system.

**Table 2:** Hierarchical linear regression on total BWAQ score.

Model		Standard Error	Standardized Coefficient	t	*p*
Model 1	BAS_Drive	0.23	0.02	0.55	0.58
	BAS_Fun Seeking	0.24	0.05	1.20	0.23
	BAS_Reward Responsiveness	0.20	−0.07	−1.18	0.24
	BIS	0.12	0.14	3.58	<0.001
Model 2	BAS_Drive	0.24	0.08	1.77	0.08
	BAS_Fun Seeking	0.25	−0.05	−1.07	0.29
	BAS_Reward Responsiveness	0.20	0.01	0.28	0.78
	BIS	0.13	0.02	0.43	0.67
	Conscientiousness	0.08	−0.15	−3.78	<0.001
	Openness to experience	0.06	0.06	1.47	0.14
	Neuroticism	0.09	0.20	4.79	<0.001
	Extraversion	0.07	0.01	0.21	0.83
	Agreeableness	0.10	0.02	0.46	0.64

BAS_Drive: motivation to follow one’s goals; BAS_Fun seeking: motivation to find novel rewards spontaneously; BAS_Reward Responsiveness: sensitivity to pleasant reinforcers in the environment. BIS: behavioral inhibition system.

**Table 3:** Hierarchical linear regression on BWAQ subscales.

		Craving				Dependency				Anticipation				Avoidance			
Model		SE	Standardized Coefficient	t	*p*	SE	Standardized Coefficient	t	*p*	SE	Standardized Coefficient	t	*p*	SE	Standardized Coefficient	t	*p*
Model 1	BAS_Drive	0.12	0.01	0.13	0.90	0.07	0.01	0.28	0.78	0.04	0.07	1.68	0.09	0.04	0.02	0.41	0.68
	BAS_Fun Seeking	0.16	0.05	1.12	0.26	0.07	0.06	1.36	0.17	0.05	0.004	0.10	0.92	0.04	0.04	0.94	0.35
	BAS_Reward Responsiveness	0.11	−0.06	−1.17	0.24	0.06	−0.002	−0.05	0.96	0.04	−0.02	−0.41	0.69	0.04	−0.12	−2.54	0.01
	BIS	0.06	0.14	3.53	<0.001	0.04	0.12	3.08	0.002	0.02	0.08	2.13	0.03	0.02	0.07	1.89	0.06
Model 2	BAS_Drive	0.13	0.06	1.24	0.22	0.07	0.07	1.51	0.13	0.05	0.11	2.31	0.02	0.04	0.05	1.04	0.30
	BAS_Fun Seeking	0.13	−0.03	−0.72	0.47	0.08	−0.05	−1.18	0.24	0.05	−0.07	−1.49	0.14	0.05	−0.01	−0.19	0.85
	BAS_Reward Responsiveness	0.11	0.02	0.32	0.75	0.06	0.06	1.17	0.24	0.04	0.01	0.20	0.84	0.04	−0.08	−1.55	0.12
	BIS	0.07	0.003	0.08	0.94	0.04	0.03	0.58	0.57	0.03	0.01	0.16	0.87	0.02	0.05	1.00	0.32
	Conscientiousness	0.04	−0.11	−2.77	0.006	0.02	−0.18	−4.40	<0.001	0.02	−0.06	−1.57	0.12	0.02	−0.14	−3.46	<0.001
	Openness to experience	0.03	0.06	1.52	0.13	0.02	0.05	1.25	0.21	0.01	0.11	2.81	0.005	0.01	−0.05	−1.43	0.15
	Neuroticism	0.05	0.22	5.12	<0.001	0.03	0.17	4.06	<0.001	0.02	0.15	3.44	<0.001	0.02	0.03	0.77	0.44
	Extraversion	0.04	−0.04	−1.08	0.28	0.02	0.07	1.76	0.08	0.01	0.03	0.62	0.53	0.01	0.03	0.64	0.52
	Agreeableness	0.05	0.01	0.18	0.86	0.03	0.04	1.18	0.24	0.02	0.06	1.68	0.09	0.02	−0.07	−1.74	0.08

BAS_Drive: motivation to follow one’s goals; BAS_Fun seeking: motivation to find novel rewards spontaneously; BAS_Reward Responsiveness: sensitivity to pleasant reinforcers in the environment. BIS: behavioral inhibition system.

## Data Availability

Data were available by the corresponding authors.
